# Therapeutic Drug Monitoring of Infliximab in Acute Severe Ulcerative Colitis

**DOI:** 10.3390/jcm12103378

**Published:** 2023-05-10

**Authors:** Benjamin L. Gordon, Robert Battat

**Affiliations:** 1 Division of Gastroenterology and Hepatology, New York-Presbyterian/Weill Cornell Medical Center, New York, NY 10065, USA; beg2013@nyp.org; 2 Center for Clinical and Translational Research in Inflammatory Bowel Diseases, Centre Hospitalier de l’Université de Montréal, Montreal, QC H2X 0A9, Canada

**Keywords:** acute severe ulcerative colitis, therapeutic drug monitoring, infliximab, pharmacokinetics, dose optimization, inflammatory bowel disease, ulcerative colitis

## Abstract

Therapeutic drug monitoring (TDM) is a useful strategy in ulcerative colitis (UC). Nearly a quarter of UC patients will experience acute severe UC (ASUC) in their lifetime, including 30% who will fail first-line corticosteroid therapy. Steroid-refractory ASUC patients require salvage therapy with infliximab, cyclosporine, or colectomy. Fewer data are available for the use of TDM of infliximab in ASUC. The pharmacokinetics of ASUC make TDM in this population more complex. High inflammatory burden is associated with increased infliximab clearance, which is associated with lower infliximab drug concentrations. Observational data support the association between increased serum infliximab concentrations, lower clearance, and favorable clinical and endoscopic outcomes, as well as decreased rates of colectomy. Data regarding the benefit of accelerated or intensified dosing strategies of infliximab—as well as target drug concentration thresholds—in ASUC patients remain more equivocal, though limited by their observational nature. Studies are underway to further evaluate optimal dosing and TDM targets in this population. This review examines the evidence for TDM in patients with ASUC, with a focus on infliximab.

## 1. Introduction

Ulcerative colitis (UC) is one of two major chronic inflammatory bowel diseases (IBD). Up to 25% of UC patients will experience an episode of acute severe ulcerative colitis (ASUC) that requires hospitalization [1,2]. Delayed management of ASUC is associated with significant morbidity and mortality, including toxic megacolon, fulminant colitis, bowel perforation, refractory bleeding, venous thromboembolism, and bacterial infection [3,4]. Colectomy rates for hospitalized patients with ASUC range from 25% to 30%, including 20% of patients requiring colectomy during their first hospital admission [1,2,5,6]. Despite the advent of biologic therapy and advances in the management of ASUC, there is still measurable mortality associated with it [6].

First-line medical therapy in patients with ASUC remains intravenous corticosteroids. However, 30% of ASUC patients will fail steroid therapy, of whom up to 50% will require colectomy [3,7,8,9]. Patients who do not demonstrate improvement within 3–5 days of initiation of corticosteroids are unlikely to respond to steroids and require either rescue therapy or colectomy [10].

Rescue therapy for ASUC includes infliximab (IFX) and intravenous cyclosporine. Multiple trials have shown that infliximab is beneficial in steroid-refractory ASUC patients, including one randomized controlled trial (RCT) which demonstrated that infliximab decreased short-term colectomy rates compared to placebo (29% vs. 67%) [11,12,13]. Cyclosporine has similarly been proven to be superior to placebo in these patients [14,15]. In multiple head-to-head RCTs of infliximab and cyclosporine in ASUC, there have been no differences in short- or long-term rates of clinical response, colectomy, or mortality [16,17]. A recent meta-analysis of trials compared rescue IFX to cyclosporine therapy in steroid-refractory ASUC [1]. In RCTs of severe UC patients receiving infliximab, pooled rates of therapeutic response, 3-month colectomy, and 12-month colectomy were 43.8%, 26.6%, and 34.4%, respectively. Similar results were observed with cyclosporine (41.7%, 26.4%, and 40.8%, respectively). In nonrandomized studies, rates were considerably higher for both infliximab (74.8%, 24.1%, and 20.7%, respectively) and cyclosporine (55.4%, 42.5%, and 36.8%, respectively) [1].

Infliximab is generally preferred over cyclosporine in ASUC patients for ease of use. Initial responders to infliximab remain on maintenance infliximab therapy. Cyclosporine requires frequent dose adjustments due to a narrow therapeutic window. In addition, responders to cyclosporine must be converted to alternate therapies, and the most robust data exist for subsequent thiopurines, which is no longer preferred as a first-line UC therapy.

For cyclosporine, data do not support intensive dosing. In a RCT of high-dose intravenous cyclosporine (4 mg/kg) versus standard dose intravenous cyclosporine (2 mg/kg), there was no significant difference in short-term colectomy rates, despite the high-dose group having significantly increased mean cyclosporine blood levels [18]. However, data regarding dose optimization and therapeutic drug monitoring (TDM) in infliximab are still being elucidated. For this reason, in this review, we will discuss TDM in ASUC, with a focus on infliximab.

## 2. Pharmacokinetics of Infliximab in ASUC

It is well understood that the clearance of anti-TNF (tumor necrosis factor) agents in ASUC is propagated by numerous mechanisms related broadly to increased inflammatory burden. The pharmacokinetics of infliximab in ASUC are impacted by increased levels of TNF-α, anti-TNF neutralization, heightened proteolytic degradation of immune complexes, and reduced tissue penetration, all of which leads to low serum concentrations of infliximab, which may subsequently augment immunogenicity [8,19,20,21,22,23,24,25]. Moreover, many ASUC patients suffer from malnutrition in the acute setting, and thus drug levels of infliximab may be negatively affected by both decreased protein intake as well as increased intestinal protein loss, resulting in hypoalbuminemia and increased infliximab clearance, as infliximab is albumin-bound [8,10,26,27].

Fecal drug loss can occur in the setting of a severely impaired mucosal barrier in patients with ASUC. Not only does this lead to lower drug levels, but it also results in effectively decreased episodic doses of infliximab, which promotes immunogenicity and antibody formation against infliximab [9]. The formation of antibodies to infliximab has ramifications on dose optimization of infliximab, and it impacts treatment decisions. In one large study using an IBD database, in infliximab-treated patients (n = 63,176) with antibodies to infliximab (ATI), dose escalation was associated with adequate infliximab levels (>5 μg/mL) at the subsequent assessment [28]. Dose escalation was also associated with a greater increase in drug concentration (5.9 vs. 0.2 μg/mL) and ATI reductions (1.9 vs. 4.3 U/mL) compared to patients with no escalation [28].

Little is known about the relationship between efficacy and serum, colonic mucosa, and stool concentrations of drugs. Mucosal 5-acetylsalicylic acid (5-ASA) concentrations are associated with endoscopic outcomes [29] but there is a paucity of data on the relationship of infliximab concentrations between tissue, serum, and fecal compartments. A recent study on TNF antagonist concentrations in colonic tissue provides minimal information on infliximab in UC. Only six patients with UC were included, of which an unspecified number received infliximab or adalimumab. Furthermore, a majority of samples analyzed in the study were from uninflamed tissue and neither clinical outcomes nor objective endoscopic scores were assessed [23]. The study demonstrated a positive correlation between serum and tissue TNF antagonist concentration in uninflamed tissue but not in inflamed tissue. Serum TNF antagonist concentrations only correlated with the degree of endoscopic inflammation in uninflamed tissue. Patients with active mucosal disease had high rates of discordant serum to tissue drug concentrations [23].

Data in ASUC are consistent with observations that infliximab concentrations are lower with higher inflammatory burden. In an observational retrospective study, Ungar et al. demonstrated that mean infliximab trough levels at day 14 were significantly lower in patients with ASUC compared to patients with moderately severe UC (7.1 vs. 14.4 μg/mL) [30] (Table 1). As ASUC impacts the pharmacokinetics of infliximab and decreases drug levels, there may be benefit to TDM in this population.

## 3. Outcomes Associated with Infliximab Pharmacokinetics in ASUC

The potential utility of TDM in ASUC initially came out of the established benefit of dose optimization in patients with moderate-to-severe UC. In a post-hoc analysis of 728 patients with moderate-to-severe UC in the ACT-1 and ACT-2 (Active Ulcerative Colitis Trials 1 and 2) trials, patients with clinical response, mucosal healing, and/or clinical remission had higher median serum concentrations of infliximab at weeks 8, 30, and 54 compared to patients without clinical or endoscopic response [31]. In a separate study of 155 patients with IBD, in patients with subtherapeutic infliximab concentrations, dose escalation led to complete or partial clinical response in 86% of patients [32]. Lastly, Brandse et al. published two separate prospective, observational studies evaluating infliximab concentrations in serum and stool in patients with moderate-to-severe UC. Primary infliximab nonresponders were found to have lower serum infliximab concentrations, increased fecal infliximab concentrations, and higher rates of antibody formation to infliximab, suggesting that higher infliximab clearance and drug wasting in the stool are associated with worse clinical outcomes [20,21].

In ASUC patients, supportive data exist showing that serum infliximab concentrations have predictive value for clinical response, clinical remission, and need for colectomy. In a prospective observational study of 115 patients with steroid-refractory acute UC (including 42 with ASUC), Seow et al. demonstrated that patients with detectable serum infliximab trough concentrations—during both induction and maintenance—had higher rates of clinical remission (69% vs. 15%) and lower rates of colectomy (7% vs. 55%) compared to patients with undetectable trough concentrations [22]. In another prospective observational study of 285 patients with refractory UC (including 39 patients with ASUC), serum levels of infliximab > 2.5 μg/mL at week 14 were associated with higher rates of relapse-free survival as well as colectomy-free survival [33]. In a retrospective study of 99 patients with UC (including 23 patients with ASUC), Papamichael et al. showed that infliximab concentration < 16.5 μg/mL at week 2 after induction was an independent predictor of colectomy [34]. Furthermore, in a study of 24 ASUC patients, elevated fecal infliximab concentrations were associated with both decreased remission rates and higher risk of colectomy [35] (Table 2).

Serum infliximab concentrations have also been associated with favorable endoscopic outcomes. For example, in Seow’s study of 115 acute UC patients (including 42 with ASUC), a detectable serum infliximab trough concentration was associated with higher rates of endoscopic improvement (decrease in endoscopic Mayo score of at least one point) compared to patients with undetectable trough concentrations (76% vs. 28%) [22]. In a prospective observational study of 52 patients with active IBD on maintenance infliximab requiring dose optimization (including 18 UC patients, 10 with severe endoscopic activity), an increase in infliximab trough level was associated with mucosal healing, and 50% of IBD patients achieved mucosal healing after dose intensification [36]. In a retrospective study of 101 UC patients (including 16 with ASUC), Papamichael et al. showed that patients with early mucosal healing (at weeks 10–14 from initiation of therapy) had higher serum infliximab concentrations at weeks 2, 6, and 14 after treatment initiation than those without healing [37]. This study also demonstrated that the presence of ASUC was associated with being in the lowest quartile of infliximab concentration among all UC patients in the study (11 of 16 ASUC patients were in the lowest quartile)—the authors of this study thus suggested that accelerated dosing be used in ASUC patients. A number of the studies outlined present their data in aggregate, including all moderate and severe UC patients, without subgroup analysis of ASUC patients. This may limit the application of these data to ASUC patients.

Clearance of infliximab is also an important determinant of pharmacokinetics and outcomes in ASUC. Higher infliximab clearance has been associated with colectomy and treatment failure. In a retrospective study of 39 patients with ASUC, elevated (>0.627 L/day) baseline calculated infliximab clearance (using sex, presence of antibodies to infliximab, and serum albumin) prior to induction was associated with higher rates of colectomy compared to patients with lower clearance values (61.5% vs. 7.7%) [27]. In another retrospective study of 36 ASUC patients, Kevans et al. demonstrated that lower clearance of infliximab (utilizing pharmacokinetic modeling of a number of parameters, including serum infliximab concentrations, antibodies to infliximab, weight, and serum albumin) was associated with week 14 clinical response and week 54 steroid-free remission [38] (Table 2).

## 4. Intensive Infliximab Dosing Strategies in ASUC

Despite evidence suggesting that increased serum infliximab concentrations and decreased infliximab clearance are associated with improved clinical and endoscopic outcomes, data regarding intensified and accelerated infliximab dosing strategies in this population are conflicting. Initial literature on intensive dosing strategies were first investigated in moderate-to-severe UC, providing the rationale for its use in ASUC. For instance, in subgroup analyses of the ACT-1 and 2 trials, a 10 mg/kg dosing strategy of infliximab significantly reduced the risk of colectomy at 54 weeks compared to placebo (8% vs. 17%), but a 5 mg/kg dosing strategy did not (12%) [39].

However, data comparing different dosing strategies of infliximab in ASUC are contradictory. Some observational data suggest a benefit to intensive infliximab induction dosing strategies in ASUC with either multiple early doses or higher doses at standard intervals. In a retrospective study of 50 ASUC patients comparing standard induction of infliximab (5 mg/kg at weeks 0, 2, and 6) to accelerated induction (3 doses of 5 mg/kg within a median period of 24 days), Gibson et al. showed that an accelerated dosing strategy was associated with lower rates of early colectomy (7% vs. 40%) [40].

Other data have not shown a benefit to intensified dosing in this cohort. In a systematic review of seven retrospective studies (181 patients receiving accelerated infliximab dosing and 436 receiving standard infliximab dosing), there were similar rates of in-hospital colectomy among the accelerated dosing group and standard dosing group (9% vs. 8%); furthermore, similar proportions required colectomy at 3, 6, 12, and 24 months [41]. In a separate meta-analysis of five observational studies, Feuerstein et al. found that there was no significant difference in short-term colectomy risk between ASUC patients given intensive infliximab dosing (shortened interval dosing during induction or higher-dose during induction) compared to standard infliximab dosing (relative risk (RR) 1.61, 95% confidence interval (CI, 0.74–3.52) [10]. Other smaller retrospective studies have similarly found no difference in short-term colectomy risk in ASUC patients receiving higher-dose induction or accelerated dosing compared to those receiving standard induction dosing [42,43,44] (Table 3). While limited data exist on different intensive dosing strategies, a meta-analysis of two observational studies found that upfront higher dose infliximab (10 mg/kg) was associated with lower rates of colectomy than accelerated dosing with 5 mg/kg (RR 0.24, 95% CI, 0.08–0.68) [10].

Interpreting observational data of intensive dosing in ASUC patients is complex. Selection bias may exist in groups with accelerated or intensified dosing for patients with higher probability to have inadequate response to standard induction therapy [10]. In addition, there may be subgroups of individuals that required personalized selective dosing that are not captured by broadly comparing two treatment strategies. Further studies comparing clearance-based dosing to standard dosing in ASUC are needed.

## 5. Specific Threshold Target Concentrations for Infliximab

Observational data have shown higher infliximab drug concentrations to be associated with clinical remission, endoscopic remission, and lower rates of colectomy [22,33,34,35,36,37]. However, the use of specific infliximab concentrations to guide therapy is complicated by several factors: ASUC patients exhibit particularly unfavorable pharmacokinetics, specific infliximab drug concentrations targets are unknown in this context, and multiple measurement timepoints and assays exist.

Despite this, indirect data for specific infliximab thresholds from moderate-to-severe UC may inform the ASUC setting. In a post-hoc analysis of ACT-1 and ACT-2 (n = 728), serum infliximab levels of 41 μg/mL at week 8 of induction were associated with clinical response (sensitivity 63%, specificity 62%, positive predictive value 80%) [31]. In a separate literature review, Chiefetz et al. identified two studies with week 2 infliximab thresholds of >11.5–15.3 μg/mL for clinical response and remission, and week 14 infliximab thresholds of >5.1–6.7 μg/mL for mucosal healing [45,46,47].

Multiple infliximab thresholds have also been evaluated in ASUC. A retrospective study of 101 UC patients (including 16 with ASUC) found that infliximab concentrations of 28.3, 15.0, and 2.1 μg/mL at weeks 2, 6, and 14, respectively, were associated with short-term mucosal healing [37]. Moreover, in a prospective observational study of 285 patients with refractory UC (including 39 patients with ASUC), Arias et al. showed that serum levels of infliximab > 2.5 μg/mL at week 14 were associated with an absence of clinical relapse (sensitivity 81%, specificity 75%) and higher rates of relapse-free survival as well as colectomy-free survival [33]. However, a smaller retrospective study of 76 patients with IBD (including 18 with UC, number of ASUC unspecified) found no significant difference in mean infliximab troughs between patients who had clinical response to intensification of infliximab compared to those who did not (3.3 μg/mL vs. 2.3 μg/mL) [48].

Expert consensus statements for TDM in IBD recommend targeting infliximab concentrations of at least 20–25 μg/mL at week 2, 15–20 μg/mL at week 6, and 7–10 μg/mL at week 14 [45]. The caveat to these recommendations is that target thresholds should be tailored to disease severity and desired therapeutic outcome, as higher drug concentrations may be needed for ASUC [45].

## 6. Maintenance Monitoring following Infliximab Salvage Therapy for ASUC

Infliximab TDM strategies post-induction are variable, and few data exist on TDM during maintenance infliximab therapy after infliximab rescue therapy for ASUC. In a small retrospective study of 41 ASUC patients, including 20 patients who were maintained on infliximab after discharge (and who had follow-up data for one year), only 4 of 20 patients (20%) had a serum infliximab level checked after discharge [49]. As a comparison, thiopurine metabolites were monitored in 15 of 27 (56%) patients [49].

To our knowledge, in adult ASUC patients, no other data exist on TDM for maintenance therapy after salvage therapy, although this has been studied prospectively in the pediatric population [50]. In this pediatric study of 38 ASUC patients receiving infliximab, higher infliximab clearance (calculated by serum albumin, ATI, and white blood cell count) was associated with lack of remission at 26 weeks from induction; furthermore, patients with clinical remission at 26 weeks had numerically—albeit not significantly—higher infliximab trough concentrations (19.5 vs. 14.2 μg/mL) [50]. The PROTOS study, “Pharmacokinetics of IFX and TNF Concentrations in Serum, Stool, and Colonic Mucosa in Acute Severe Ulcerative Colitis”, is an ongoing open-label, prospective, observational study to better assess the pharmacokinetics of infliximab in adult ASUC patients in the acute and maintenance setting [51]—studies such as this will potentially provide better data regarding timing of TDM and drug concentration thresholds of this cohort during the induction and maintenance period.

## 7. Cost-Effectiveness of TDM of Infliximab in ASUC

The use of TDM of infliximab has not only proven to be a useful strategy in IBD, but it has also been shown to be cost-effective. In one prospective observational multicenter study of 96 IBD patients with loss of response to infliximab managed according to a TDM algorithm compared to 56 historical controls treated empirically with dose intensification, there were similar rates of clinical response at 12 weeks. However, patients managed with TDM were less likely to have infliximab dose escalations and by cost analysis there was an estimated 15% savings with the TDM algorithm [52]. A separate systematic review identifying two RCTs (including 247 Crohn’s disease patients and 85 UC patients) found that the cost savings from TDM dosing strategies ranged from 28% to 34% [53]. However, while the use of infliximab in ASUC has been demonstrated to be cost-effective compared to both cyclosporine and surgery [54], to our knowledge, there have been no studies of cost-effectiveness of TDM of infliximab in ASUC patients. This remains an area for future research.

## 8. Conclusions and Future Directions

The pharmacokinetics of infliximab are altered in the severely inflamed state of ASUC, leading to lower drug concentrations and higher clearance. Observational data show that lower infliximab levels and higher clearance are associated with worse symptoms, more colonic inflammation, and higher rates of colectomy. However, observational studies on the use of intensive dosing strategies to overcome lower infliximab concentrations in ASUC are equivocal.

Based on the reported pharmacokinetics of infliximab in ASUC, very high doses of infliximab are likely to be required to induce clinical and endoscopic responses. In addition, there are inter-individual differences in infliximab clearance between ASUC patients. Thus, some ASUC patients may benefit from intensive dosing strategies, while others only require standard dosing. Determining the optimal dosing strategy for each patient in a personalized manner would likely lead to improved outcomes. However, to date there are no trials comparing clearance-based dosing strategies to standard dosing.

Besides robust data supporting TDM strategies in ASUC, another potential obstacle to the broader adoption of TDM is cost and time lag between sample collection and results. In a survey of 403 gastroenterologists and their attitudes towards TDM of anti-TNF agents in IBD, the largest barriers to widespread TDM implementation were perceived to be insurance coverage (78%), out-of-pocket costs (76%), and lag time between sample collection and result (39%) [55]. Point-of-care assays for TDM exist [56] and should be further explored in ASUC to address time lag concerns. In addition, further cost-effectiveness studies may further impact payor decisions to support TDM in ASUC.

Two ongoing clinical trials will hopefully provide answers to some of these unmet questions. PREDICT UC or “Optimising Infliximab Induction Therapy for Acute Severe Ulcerative Colitis” is a multicenter RCT investigating whether accelerated dose infliximab (5 mg/kg at weeks 0, 1, and 3) or higher-dose infliximab (10 mg/kg at weeks 0 and week 1) is superior to standard dose infliximab (5 mg/kg at weeks 0, 2, and 6) in improving clinical response and decreasing short-term colectomy rates [57]. The study was completed in September 2022, and data should become available soon. Additionally, TITRATE, or inducTIon for acuTe ulceRATivE Colitis, is a multicenter RCT evaluating whether proactive individualized intensified infliximab dosing in ASUC patients—using a pharmacokinetics-driven dashboard system—can lead to better clinical and endoscopic responses at week 6 compared to standard dosing [58]. This study is planned to be completed in December 2024. Further studies will clarify the use of TDM in ASUC patients and potentially improve outcomes in this population.

## Figures and Tables

**Table 1:** Summary of Pharmacokinetic Studies of Infliximab in ASUC.

Author (Year)	Study Design	Population	Number of Subjects	Measurement of IFX Pharmacokinetics	Outcomes
Yarur (2016) [23]	Prospective Cross-Sectional	IBD patients on maintenance IFX or adalimumab	30 (including 6 UC)	Anti-TNF serum concentration and anti-TNF tissue concentration (from colonic and ileal biopsies)	Anti-TNF tissue concentrations correlated with anti-TNF serum concentrations, except in inflamed tissue Ratio of anti-TNF to TNF in tissue was highest in uninflamed tissue and lowest in severely inflamed tissue
Dotan (2014) [25]	Prospective Observational	IBD patients receiving IFX	54 patients (25 UC, 25 Crohn’s disease, and 4 indeterminate) with 169 IFX concentrations	IFX trough concentrations and antibodies against IFX prior to IFX infusion	Low albumin, high body weight, and presence of antibodies against IFX were associated with higher IFX clearance
Fasanmade (2009) [26]	Post-hoc analysis of 2 RCTs (ACT 1 and 2)	Moderate-to-severe UC randomized to IFX 5 mg/kg or 10 mg/kg or placebo	482	IFX serum concentrations immediately before and after IFX doses and antibodies against IFX	IFX clearance was higher in patients with antibodies to IFX IFX clearance was inversely correlated with serum albumin
Brandse (2016) [20]	Prospective Cohort	Anti-TNF naïve moderate–severe UC patients receiving IFX induction therapy	19	Serial IFX serum concentrations and antibodies against IFX	IFX nonresponders were more likely to have antibodies against IFX (odds ratio 30.0, 95% CI 2.2–406) Patients with CRP > 50 mg/L at baseline had lower serum IFX concentrations at week 6 compared to patients with lower CRP
Brandse (2015) [21]	Prospective Cohort	Anti-TNF naïve patients with moderately to severely active UC, initiated on IFX	30 (26 with severe endoscopic disease)	IFX serum concentration at week 2 and IFX fecal concentration at day 1 from induction	Clinical nonresponders at week 2 had significantly increased fecal IFX levels at day 1 from induction compared to responders (5.01 vs. 0.54 μg/mL)
Ungar (2016) [30]	Retrospective	Hospitalized steroid-refractory ASUC patients compared to moderately severe UC initiated on IFX	32 total (including 16 ASUC)	IFX trough concentrations and antibodies to IFX at day 14 from induction	IFX trough concentrations were significantly lower in ASUC patients compared to moderately severe UC patients (7.2 vs. 14.4 μg/mL)

**Table 2:** Summary of Outcomes Associated with Infliximab Pharmacokinetics in ASUC.

Author (Year)	Study Design	Population	Number of Subjects	Measurement of IFX Pharmacokinetics	Outcomes Associated with IFX Pharmacokinetics
Seow (2010) [22]	Prospective Observational	Steroid-refractory acute moderate-to-severe UC patients initiated on IFX	115 total (including 42 ASUC)	Detectable serum IFX trough concentration during induction and maintenance periods (found in 39% of subjects)	Higher rates of clinical remission (69% vs. 15%), lower rates of colectomy (7 vs. 55%), and higher rates of endoscopic improvement (76% vs. 28%) were found in patients with detectable troughs compared to those with undetectable troughs
Arias (2015) [33]	Prospective Observational	UC patients refractory to cyclosporine or immunomodulators, initiated on IFX	285 total (including 39 ASUC)	Serum IFX level at week 14 of treatment	Serum IFX level > 2.5 μg/mL at week 14 was associated with increased rates of relapse-free survival and colectomy-free survival
Papamichael (2016) [34]	Retrospective Observational Multicenter	Anti-TNF naïve UC patients with primary nonresponse to IFX induction therapy	99 total (including 23 ASUC)	Serum IFX levels at weeks 2 and 6 of treatment	Serum IFX level < 16.5 μg/mL at week 2 (hazard ratio 5.6, 95% CI 1.1–27.8) was independently associated with colectomy
Beswick (2018) [35]	Prospective Observational Pilot	Hospitalized steroid-refractory ASUC patients, initiated on IFX	24 total (all 24 with ASUC)	Fecal IFX concentration at day 1 post-first dose of IFX	Fecal IFX level > 1 mg/mL at day 1 was associated with lower rates of clinical remission at week 6 (odds ratio 0.04, 95% CI 0.02–0.9) and higher rates of colectomy (odds ratio 176, 95% CI 2.1–14,452)
Paul (2013) [36]	Prospective Observational	IBD patients receiving IFX who required IFX dose optimization for active disease	52 total (18 UC, including 10 ASUC)	IFX trough concentrations prior to IFX optimization and at week 8 after optimization; differences in trough concentrations were calculated (called delta IFX)	Delta IFX > 0.5 μg/mL was associated with mucosal healing (sensitivity 0.88, specificity 0.77)
Papamichael (2016) [37]	Retrospective	UC patients receiving IFX induction	101 total (including 16 ASUC)	Serum IFX levels at weeks 2, 6, and 14 after induction	Early mucosal healing was associated with increased serum IFX levels at weeks 2 (22.9 vs. 19.3 μg/mL), 6 (17.6 vs. 10.3 μg/mL), and 14 (7.4 vs. 1.5 μg/mL) compared to those without healing
Battat (2021) [27]	Retrospective	Hospitalized ASUC patients, initiated on IFX	39 total (all 39 with ASUC)	Baseline calculated IFX clearance using existing formula (that included sex, presence of antibodies to IFX, and serum albumin)	IFX clearance > 0.627 L/day was associated with higher rates of colectomy within 6 months compared to those with lower clearance (61.5% vs. 7.7%)
Kevans (2018) [38]	Retrospective	Steroid-refractory ASUC patients, initiated on IFX	36 total (all 36 with ASUC)	IFX clearance using pharmacokinetic modeling (that included serum IFX levels, antibodies to IFX, weight, and serum albumin)	Lower IFX clearance was associated with higher rates of clinical response at week 14 and steroid-free remission at week 54

**Table 3:** Summary of Intensive Dosing Strategy Studies in ASUC.

Author (Year)	Study Design	Population	Number of Subjects	Intensive Dosing Strategy	Primary Outcome	Results
Gibson (2015) [40]	Retrospective Cohort	Hospitalized patients receiving IFX for steroid-refractory ASUC	50 total (n = 35 standard dosing; n = 15 accelerated dosing	Three induction doses of IFX 5 mg/kg in median 24 days	Colectomy during IFX induction	Significantly decreased rates of early colectomy in the accelerated arm (7% vs. 40%)
Shah (2018) [42]	Retrospective Cohort with Propensity Score Matching	Hospitalized, IFX-naïve, acute UC patients receiving induction IFX	146 total (n = 120 standard dose; n = 26 high dose)	10 mg/kg induction dose of IFX	30-day colectomy	No significant difference in 30-day colectomy rates between high dose and standard dose groups in the unmatched cohort (15.4% vs. 17.5%) and matched cohort (9.5% vs. 9.5%)
Chao (2019) [43]	Retrospective Cohort	Hospitalized ASUC patients receiving IFX	72 total (n = 37 standard dose induction; 35 high dose induction)	10 mg/kg induction dosing of IFX	Three-month colectomy	No significant difference in three-month colectomy rates between high dose and standard dose groups (14.3% vs. 5.4%)
Govani (2020) [44]	Retrospective Cohort	Hospitalized ASUC patients receiving IFX	66 total (n = 33 standard dosing; 33 accelerated dosing)	Two doses of IFX prior to day 14	90-day colectomy	No significant difference in 90-day colectomy rates between accelerated dosing and standard dosing groups (30.3% vs. 24.2%)
Nalagatla (2019) [41]	Retrospective Cohort and Meta-analysis of 7 Retrospective Studies (3 full text, 4 abstract)	Hospitalized patients receiving IFX for steroid-refractory ASUC	Retrospective Cohort: 213 total (n = 132 standard dosing; n = 81 accelerated dosing) Meta-analysis: 617 total (n = 436 standard dosing; n = 181 accelerated dosing)	10 mg/kg induction dosing of IFX or 5 mg/kg dosing at intervals shorter than weeks 0, 2, and 6	Retrospective Cohort: in-hospital colectomy Meta-analysis: in-hospital colectomy or one-month colectomy	No significant difference in in-hospital colectomy between accelerated dosing and standard dosing groups (9% vs. 8%) No significant difference in early colectomy between accelerated dosing and standard dosing in the meta-analysis (odds ratio 0.76, 95% CI 0.36–1.61)
Feuerstein (2020) [10]	Meta-analysis of 5 Observational Studies	Hospitalized patients receiving IFX for steroid-refractory ASUC	Total subjects not given	Shortened interval between IFX dosing (<2 weeks, dose stacking) or 10 mg/kg induction dosing	Short-term risk of colectomy	No significant difference in short-term risk of colectomy between intensive and standard dosing groups (relative risk 1.61, 95% CI 0.74–3.52)

## Data Availability

No new data were created or analyzed in this study. Data sharing is not applicable to this article.
